# Private Mitochondrial DNA Variants in Danish Patients with Hypertrophic Cardiomyopathy

**DOI:** 10.1371/journal.pone.0124540

**Published:** 2015-04-29

**Authors:** Christian M. Hagen, Frederik H. Aidt, Ole Havndrup, Paula L. Hedley, Morten K. Jensen, Jørgen K. Kanters, Tam T. Pham, Henning Bundgaard, Michael Christiansen

**Affiliations:** 1 Department of Congenital Disorders, Statens Serum Institut, Copenhagen, Denmark; 2 Department of Biomedical Sciences, University of Copenhagen, Copenhagen, Denmark; 3 Department of Cardiology, Roskilde Hospital, Roskilde, Denmark; 4 Department of Medicine B, The Heart Center, Rigshospitalet, Copenhagen, Denmark; San Francisco Coordinating Center, UNITED STATES

## Abstract

Hypertrophic cardiomyopathy (HCM) is a genetic cardiac disease primarily caused by mutations in genes coding for sarcomeric proteins. A molecular-genetic etiology can be established in ~60% of cases. Evolutionarily conserved mitochondrial DNA (mtDNA) haplogroups are susceptibility factors for HCM. Several polymorphic mtDNA variants are associated with a variety of late-onset degenerative diseases and affect mitochondrial function. We examined the role of private, non-haplogroup associated, mitochondrial variants in the etiology of HCM. In 87 Danish HCM patients, full mtDNA sequencing revealed 446 variants. After elimination of 312 (69.9%) non-coding and synonymous variants, a further 109 (24.4%) with a global prevalence > 0.1%, three (0.7%) haplogroup associated and 19 (2.0%) variants with a low predicted *in silico* likelihood of pathogenicity, three variants: *MT-TC*: m.5772G>A, *MT-TF*: m.644A>G, and *MT-CYB*: m.15024G>A, p.C93Y remained. A detailed analysis of these variants indicated that none of them are likely to cause HCM. In conclusion, private mtDNA mutations are frequent, but they are rarely, if ever, associated with HCM.

## Introduction

Hypertrophic cardiomyopathy (HCM) is an inherited heart disease with a prevalence of 1:500 in the general population [1]. It is defined by hypertrophy of the left ventricle in the absence of other cardiac or systemic causes (e.g. systemic hypertension or aortic valve stenosis). HCM patients exhibit syncope, dyspnea, anginal pain and palpitations and around 10% develop progressive heart failure. A propensity to ventricular arrhythmias makes HCM the most frequent cause of sudden cardiac death in young adults [2]. However, the clinical presentation is heterogeneous and variable both between and within families suggesting the presence of modifying genes or gene-environment interaction [1,3]. More than 1400 mutations associated with HCM have been identified in 54 genes [1,4–8]. The majority of these genes encode sarcomere or sarcomere-associated proteins. Despite the considerable number of genes the genetic etiology can only be established in ~60% of the cases using current technology [9]. When a causative mutation is identified in an index case, cascade screening of the family is possible and may be of clinical significance, particularly in children [10].Mitochondria have several essential functions of particular cardiac relevance, including synthesis of adenosine triphosphate (ATP) via oxidative phosphorylation (OXPHOS), calcium signaling, generation of reactive oxygen species (ROS) and apoptosis [9,11,12]. In patients with mitochondrial disease cardiomyopathy is present in up to ~40%, either as part of multi-organ syndrome or as the only presenting feature [9]. The pathogenic mechanism underlying HCM in mitochondrial disease is believed to be abnormal OXPHOS function, i.e. perturbed synthesis of ATP and ROS [13].Mitochondrial DNA (mtDNA) is a circular DNA of ~16.6 kb. It contains 37 genes; 13 mRNAs coding for subunits of OXPHOS and 22 tRNAs and 2 rRNAs needed for intra-mitochondrial translation. The remainder of the mitochondrial proteome, consisting of over 1,000 proteins, is encoded by genes of the nuclear DNA [14]. The mitochondrial genome is characterized by 1) being maternally inherited without recombination, 2) having a high mutation rate, and 3) being subject to replication independent of the cell cycle. These characteristics may lead to the accumulation of different mtDNA molecules in a cell or tissue. This phenomenon, heteroplasmy, may result in mutated mtDNA accumulating to a level where it interferes with the function of a specific tissue. The threshold for such an effect of heteroplasmy varies, but is lowest in the heart and the brain, due to the high energy consumption in these organs [15]. The potential significance of mtDNA mutations at the population level is illustrated by the 1: 200 prevalence of ten common pathogenic mtDNA mutations in a British population, although the heteroplasmy proportion was below the phenotypic threshold [16]. Additionally, a study performed in the North East of England, found a prevalence of 9.2 in 100,000 of clinically manifest mtDNA disease, indicating that mtDNA disease is one of the most common inherited neuromuscular disorders [17].

Groups of evolutionary related mtDNA genotypes (haplogroups), are associated with differences in OXPHOS function and ROS production [18,19]. The haplogroup variation has been shown to confer differences in penetrance or expression of clinical cardiovascular phenotypes: i.e. transient ischaemic attack and ischaemic stroke [20], as well as ischemic cardiomyopathy [21], HCM [22–24], idiopathic dilated cardiomyopathy [25] and cardiomyopathy in combination with known disease causing nuclear DNA mutations [26]. It has been suggested that the association is the result of the occurrence of specific rare “private” variants in mtDNA belonging to a specific haplogroup [27]; and that younger haplogroups would not have had time to eliminate such variants through evolutionary selection pressure [28,29]. A recent comprehensive analysis of 7000+ mtDNA genomes in 11 diseases [30] clearly demonstrated that mtDNA variants could exhibit protective and susceptibility effect in several diseases simultaneously, and that the number of susceptibility variants outnumber the protective variants. Thus, the mitochondrial genome is still under strong selective pressure.Thus, mtDNA mutations can act as primary disease causing mutations or as modifiers in combination with either pathological genetic variants or environmental factors. In consequence, it is challenging to predict whether a new or rare mtDNA variant is disease associated [31–36]. This is underscored by the many erroneous conclusions regarding the classification of specific variants that have appeared over time [37–39]. This problem can be counteracted through establishing strategies and guidelines for the correct identification of pathogenic mtDNA mutations [29,31,33,35,40,41]. The purpose of this study was to ascertain the significance of private, non-haplogroup specific, mtDNA variants in a consecutively recruited cohort of Danish patients with HCM.

## Materials and Methods

### Ethics statement

All patients gave written informed consent and the study was approved by the ethics committee of Copenhagen and Frederiksberg (KF V92213).

### Study population

Eighty-seven unrelated consecutively diagnosed HCM patients identified at, or referred to, Copenhagen University Hospital, Rigshospitalet, Copenhagen, Denmark. All patients have been previously described [22]. In short, patients were subjected to a full clinical evaluation including family history, physical examination, echocardiography and ECG. All fulfilled classical diagnostic criteria for familial HCM [42,43]. The mean age of index patients was 49 years, 62% were male, and 48% of cases were familial (Table 1). All patients had been screened for mutations in the coding regions of *MYH7*, *MYBPC3*, *TTNT2*, *TPM1*, *TNNI3*, *MYL3*, *MYL2*, *ACTC*, *TCAP*, *CSRP3*, *CRYAB*,*KCNE1-5*, *GLA* and exons 3,7,14,18, and 49 of *TTN*, as detailed in previous studies [7, 44, 45]. In 32 index patients putative disease-causing mutations were identified, i.e. 11 in *MYH7*, 8 in *MYBPC3*, 2 in each of *TNNT2*, *TNNI3* and *GLA*, 1 in each of *ACTC*, *TPM1*, *MYL3* and *MYL2*. Two patients were carriers of mutations in both *MYL2* and *MYH7*.

**Table 1:** Demographic and clinical characteristics of HCM probands.

Parameter	
Age (years)*	**49 (16)**
Male/female (ratio)	**54/33**
BP systolic (mmHg)*	**126 (25)**
BP diastolic (mmHg)*	**76 (14)**
LA (mm)* 1	**45 (10)**
Max LVD (mm) * 1	**21 (6)**
MaxIVS(mm)* 1	**20 (5)**

*Mean (SD).

^1^ In index patients > 18 years of age. BP, blood pressure; LA, left atrial diameter; MaxLVD, maximal left ventricular wall thickness: MaxIVS, maximal interventricular wall thickness.

#### Sequencing of mtDNA

DNA was extracted from blood using the Maxwell 16 System (*Promega Corporation*, *Madison*, *Wisconsin*, *USA*). Forty-six PCR primer sets were designed using the Reversed Cambridge sequence (rCRS, GenBank ID: NC_012920) to cover the whole human mtDNA (Amplicon sizes 312–578 bp.). All PCR primer sets were analysed using Primer-BLAST (Database = Genome (reference assembly from selected organisms), Organism = Homo sapiens) to exclude primer sets with possible co-amplification of nuclear mitochondrial DNA. All PCR reactions were performed at an annealing temperature of 60°C. All primer sequences are available on request. The PCR products were sequenced using BigDye Terminator v1.1 Cycle Sequencing Kit (*Applied Biosystems*, *Life Technologies Corporation*, *Carlsbad*, *California*, *USA*), and analysed on an ABI3730 DNA Analyzer. The resulting sequences were aligned and compared to the rCRS using Sequencher 5.0 software (*Gene Codes*, *Ann Arbor*, *USA*). Variants were called only if observed in both forward and reverse direction. The Sanger sequencing reads were scrutinized for evidence of low-heteroplasmy of mtDNA mutations previously associated with HCM [6]. This method allows for detection of ≥20% heteroplasmy [46].

### Construction of Index6, a combined in silico algorithm predicting pathogenicity of mtDNA variants in protein coding genes

A combined algorithm for prediction of pathogenicity was constructed by obtaining the scores of 27 clearly pathogenic mtDNA variants (confirmed (Cfrm) pathogenic status in the MitoMap database [6]) and 77 neutral mtDNA variants (GenBank frequency >1% and no disease association in the MitoMap database [6]). Scores were obtained using the prediction algorithms SIFT (sift.jcvi.org/), PolyPhen2 (genetics.bwh.harvard.edu/pph2/), MutationAssessor (mutationassessor.org/), A-GVGD (http://agvgd.iarc.fr/agvgd_input.php), Provean (http://snps.biofold.org/snps-and-go/pages/method.html) SNAP (https://rostlab.org/services/SNAP/), MutPred (http://mutpred.mutdb.org/), Phd-SNP (http://snps.biofold.org/phd-snp/phd-snp.html), PANTHER (http://www.pantherdb.org/tools/csnpScoreForm.jsp) and SNP&GO (http://snps.biofold.org/snps-and-go/pages/method.html). Using these scores an optimized combination, Index6, was constructed through backward linear regression. The optimized Index6 = 0.4487*PolyPhen2-score + 0.6225*Phd-SNP-score + 0.0019*Grantham Difference, exhibited a specificity of 0.97 and sensitivity of 0.80 at an Index6 cutoff score of 0.75.

### Evaluation of mtDNA variants

**Fig 1:**
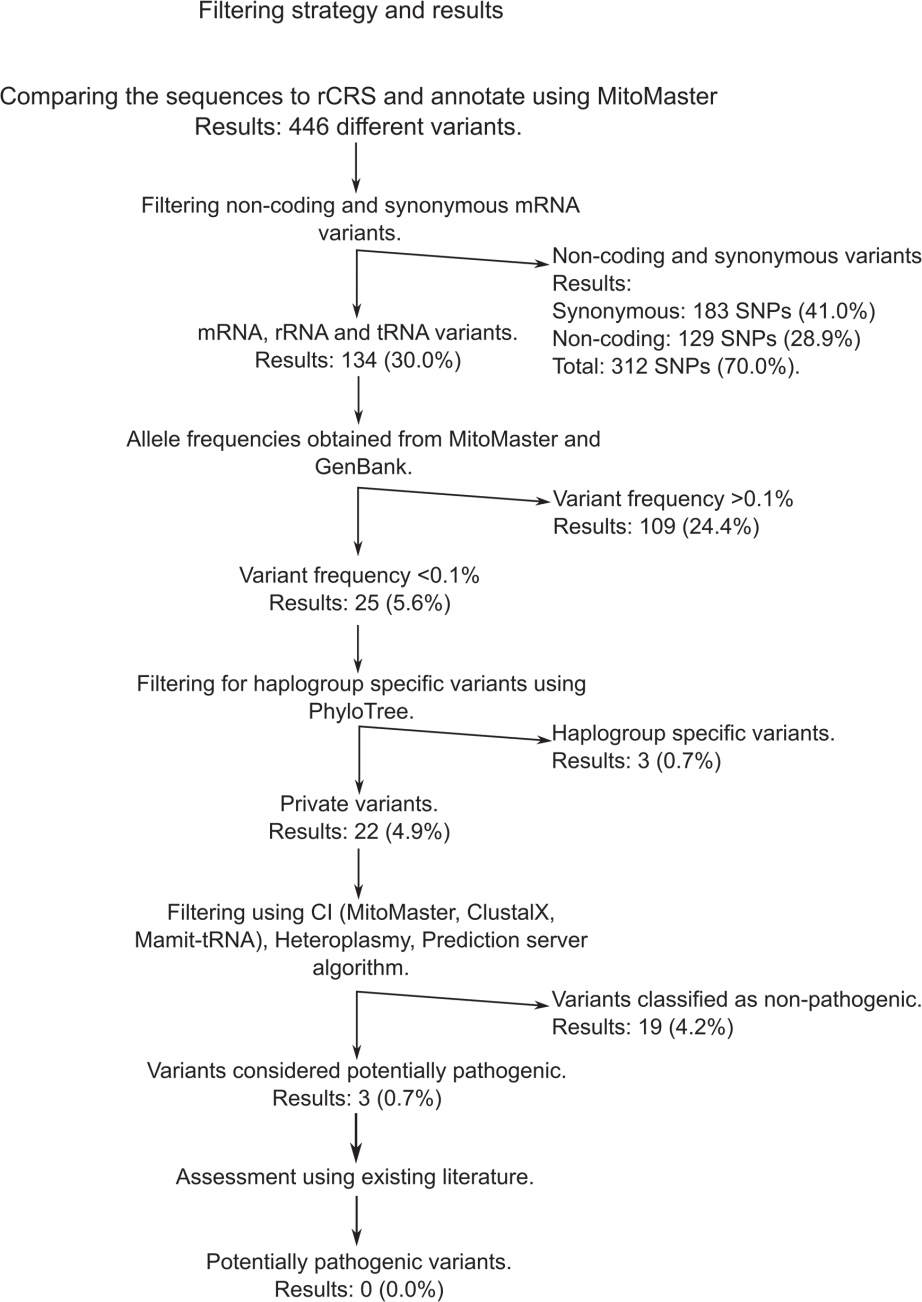
The algorithm used to select for potentially pathogenic mtDNA variants.

In order to identify potential HCM associated mtDNA variants we performed a multi-step selection procedure as depicted in Fig 1. In the first step synonymous and non-coding variants were identified using MitoMaster (www.mitomap.org/MITOMASTER) and removed. In step two, the global allele frequencies were obtained using MitoMaster and GenBank (www.ncbi.nlm.nih.gov/genbank/) and variants with a frequency >0.1% were removed. In step three, any haplogroup association for remaining variants was established using PhyloTree Build 16 (Feb 2014) [47] and they were removed if they were part of the variants defining the mtDNA haplogroup of the patient. Thereafter, mtDNA variants in protein and RNA coding genes were evaluated separately.

### Evaluation of variants in protein coding genes

Evaluation of variants in protein coding genes was based on the conservation of the physico-chemical properties of the involved amino acid residue (ClustalX), the conservation of nucleotide (MitoMaster), and *in silico* predicted functional significance using Index6. The following species were chosen for conservation analysis using ClustalX (www.clustal.org) and Jalview (www.jalview.org): *Homo sapiens*, *Pan Troglodytes*, *Bos Taurus*, *Gadus Morhua*, *Gallus Gallus*, *Mus Musculus Musculus*, *Strongylocentrotus Purpuratus*, *Xenopus Laevis*, *Neurospora Crassa and Saccharomyces Cerevisiae*. A conservation index (CI) of 10 (+) was considered highly conserved, CI≥8 conserved and CI<8 not conserved. Variants with a score of CI≥8 and an Index6 score ≥0.75 were considered potentially pathogenic and was further assessed using the existing literature.

### Variants in tRNA coding genes

Transfer-RNA (tRNA) variants were evaluated based on the gene affected (and the known disease associations of variants in this gene), on the structural significance of position and the conservation of the nucleotide using MitoMaster and Mamit-tRNA (http://mamit-trna.u-strasbg.fr/). The conservation of nucleotides was inferred from the statistical base paring in the tRNAs from the clade Euarchontoglires (Supraprimates), consisting of 34 organisms. 100% was considered highly conserved, ≥95% conserved, <95% not conserved. Highly conserved were considered potential pathogenic and was further assessed using available information on structure-function and prior clinical associations.

## Results

In the 87 individuals sequenced we identified at total of 446 different variants compared to the rCRS. The distribution of the 446 variants among the mtDNA genes is summarized in Table 2. 129 (28.9%) variants in non-coding regions and 183 (41.0%) synonymous variants were deselected, leaving 134 (30.0%) non-synonymous variants after first filtering step. This number was reduced to 25 (5.6%) after removing variants with a frequency >0.1%. Of the remaining 25 single nucleotide variants (SVNs), two belonged to the mtDNA haplogroup of the proband and two (*MT-ATP6*:m.8881T>C,p.S119P and *MT-CO1*:m.6072A>G,p.I157V) were novel. The average number of private variants per individual was one. No evidence of heteroplasmy was found for the previously HCM-associated mtDNA mutations *MT-TL1*: m.3260A>G, *MT-TL1*: m.3303C>T, *MT-T1*:m.4300A>G, *MT-T1*:m.4295A>G, *MT-TK*: m.8348A>G, *MT-TG*:m.9997T>C, *MT-CYB*:m.15243G>A and *MT-CYB*:m.15495G>A [6].

**Table 2:** The gene distribution of the 446 identified variants in the mtDNA genomes.

Region	Locus	Number of variants	Percent
Non-coding	*MT-HV1*, *MT-HV2*, *MT-HV3*, *MT-NC7*, *MT-NC5*, *MT-7SDNA*.	129	28.9
Transfer-RNA	*MT-TF*, *MT-TQ*, *MT-TC*, *MT-TY*, *MT-TS1*, *MT-TD*, *MT-TK*, *MT-TG*, *MT-TR*, *MT-TH*, *MT-TT*	21	4.7
Ribosomal-RNA	*RNR1*, *RNR2*	26	5.8
Coding	*MT-ND1*, *MT-ND2*, *MT-CO1*, *MT-CO2*, *MT-ATP6*, *MT-ATP6*, *MT-COIII*, *MT-ND3*, *MT-ND4L*, *MT-ND4*, *MT-ND5*, *MT-ND6*, *MT-CYB*	270	60.5

### Analysing the variants

A total of 25, five tRNA and 20 mRNA variants, remained after the first two steps of selection and were further analysed using standard criteria (Table 3). Three variants: *MT-TC*: m.5772G>A, *MT-TF*: m.644A>G, and *MT-CYB*: m.15024G>A, p. C93Y, fulfilled the criteria for being considered as potential pathogenic and were further assessed using the existing literature.

**Table 3:** Private variants with a GenBank frequency < 0.1%.

Locus	rCRS position	Frequency	Haplogroup	Frequency GenBank	Consequence	Mitomap status	Conservation (MitoMaster)	Conservation ClustalW	Marmit-trna (Euarchontoglires)	Patient ID. (Haplogroup)	Index score	Sarcomeric mutation
MT-TF	m. 644A>G	1	D4h4a, M38b	0.05	70A:3T WC-> G:T wobble	P	84.4%	N/A	Highly conserved	M(H1n1a)		MYH7, Arg1475Cys
MT-ND1	m.3746C>T	1	none	0.02	A147V	P	88.8%	Conserved (8)	N/A	XH (H5a2)	0.59	TNNT2, Asp286His
MT-ND2	m.5427A>G	1	None	0.01	T320A	-	60.0%	Not Conserved (0)	N/A	J (X2b6)	0.28	none
MT-TC	m.5772G>A	1	None	0.04	62C:52G WC > T:G wobble	P	2.2%	N/A	Highly conserved	ZJ (E1b)		MYBPC3, Asp230Asn
MT-CO1	m.6072A>G	1	None	0.00	I57V	-	77.7%	Conserved (9)	N/A	Y (H16)	0.22	none
MT-CO1	m.6445C>T	1	T2d2	0.04	p.T181M	N/A	95.6%	Not Conserved (6)	N/A	ZF(T1a1)	0.11	MYBPC3, InsG15919
MT-TS1	m.7473A>G	1	None	0.02	nt. 45	-	77.8%	N/A	Not Conserved	Y (H16)		none
MT-CO2	m.8084A>G	1	N1b1a1	0.03	T167A	P	13.3%	Not conserved (7)	N/A	GEC(T2b)	0.09	none
MT-ATP6	m.8573G>A	1	I1c, N1a1a1b	0.08	G16D	P	93.3%	Not conserved (5)	N/A	ZU(H7)	0.63	none
MT-ATP6	m.8645A>G	1	H1c13	0.06	N40S	P	95.6%	Conserved (9)	N/A	ZD (H3h)	0.74	none
MT-ATP6	m.8666A>G	1	None	0.02	Q47R	-	73.3%	Not Conserved (2)	N/A	F (J1c8a1)	0.24	none
MT-ATP6	m.8881T>C	1	None	0.00	S119P	-	4.4%	Not Conserved (1)	N/A	ZI (V1a1)	0.63	MYBPC3, Glu258Lys
MT-ATP6	m.9041A>G	1	J1d6, M68a2a	0.07	H172R	N/A	88.9%	Not conserved (4)	N/A	YG(V7a)	0.73	none
MT-ATP6	m.9101T>C	1	H30b1,R1a1b, U2e1f1	0.07	I192T	P/LHON, reported	13.3%	Not conserved (5)	N/A	XY(H1)	0.03	none
MT-ND4	m.10863G>A	1	None	0.01	S35N	-	91.1%	Not conserved (6)	N/A	XL (U5b2b4)	0.89	none
MT-ND5	m.14122A>C	1	None	0.02	I596L	-	24.4%	Not conserved (5)	N/A	XN (D4b1a2a1)	0.14	none
MT-CYB	m.14751C>T	1	B4a4	0.06	T2I	P	80.0%	Not conserved (0)	N/A	F(J1c8a1)	0.22	none
MT-CYB	m.14813A>G	1	None	0.01	T23A	-	55.6%	Not conserved (5)	N/A	XF (H11a)	0.06	none
MT-CYB	m.15024G>A	1	F1e	0.05	C93Y	P/Possible DEAF modifier	97.8%	Conserved (8)	N/A	B(U5b2a3)	1.06	MYH7, Arg1712ys
MT-CYB	m.15482T>C	1	None	0.02	S246P	p	66.7%	Not conserved (5)	N/A	I (H3)	0.66	none
MT-CYB	m.15813T>C	1	None	0.07	V356A	P	24.4%	Not conserved (7)	N/A	YF (U5a1c2a)	0.29	none
MT-TT	m.15894G>A	1	J1d3a	0.06	7G:66C WC-> A:C mismatch	P	84.4%	N/A	Not Conserved	ZK(H1n1)		none

#### Transfer RNA variants

The two highly conserved tRNA variants *MT-TC*: m.5772G>A and *MT-TF*: m.644A>G, both results in a change from Watson-Crick base-paring to wobble base pairing. The thermodynamic stability of wobble base paring is comparable to that of Watson-Crick base paring and the change is not expected to influence the helical structure [48,49]. In addition, these base pairs are not involved in tertiary interactions or are post-transcriptionally modified [50,51]. The variants were identified in patients, M and ZJ, where putative disease causing sarcomeric mutations already have been identified (Table 3). In consequence, *MT-TC*: m.5772G>A, and *MT-TF*: m.644A>G, are considered to be of unknown significance, but without any strong evidence in favour of pathogenicity.

#### mRNA variants

All of the mRNA variants, except one *MT-CYB*: m.15024G>A, p. C93Y, were assessed to be of no clinical significance (Table 3). *MT-CYB*: m.15024G>A has previously been described both as a potential modifying mutation, based on molecular modelling, in this cohort, and as a potential DEAF modifier [36,52].

## Discussion

The purpose of this study was to examine the mitochondrial genome of 87 well characterized HCM probands for private variants and to apply an *in silico* strategy to identify potentially pathogenic private mtDNA mutations. We identified 446 different variants of which three, *MT-TC*: m.5772G>A, *MT-TF*: m.644A>G, and *MT-CYB*: m.15024G>A, p. C93Y (~0.7%) remained after the multi-step analysis.

These findings are in concordance a previous study where a similar selection strategy was applied on 270 variants identified in 29 mtDNA sequences from Italian patients with mitochondrial cardiomyopathies [29]. Six of these (2.2%) were found to be novel non-synonymous variants and two (0.7%) were considered to be potentially pathogenic [29].

Haplogroups confer different functional characteristics on mitochondrial function as shown in several studies [18,19,53]. Thus, mtDNA haplogroup associated variants can have functional significance either as single variants or in combination with other haplogroup associated variants and/or nuclear DNA mutations/haplotypes. Recently, it has been reported that the homoplasmic mutation *MT-TH*: m.12192G>A which is a haplogroup defining variant for B5b1a3, G2a5, H45a and L3e1b3 strongly predisposes for development of HCM and dilated cardiomyopathy [54], despite a global prevalence of 0.24 (Table 4). This emphasizes that the selection strategy applied here could deselect mutations with low penetrance as, for example, the LHON associated *MT-ND6*: m.14484T>C, p.M64V, an incidental finding in one of HCM probands (Data not shown). The variant has recently been suggested to be a haplogroup specific variant for Q3b and M81 [47, 55].

**Table 4:** Variants previously associated with HCM.

Locus	rCRS position	Haplogroup	Freq GB	Consequence	Mitomap status	Conservation (MitoMaster)	Conservation ClustaW	Marmit-trna (Euarchontoglires)	Patient ID. (Haplogroup)	Index6 score
MT-RNR1	m.869C>T	L2c2a1, E1b	0.1	nt. 222 C>T	R/found in 1 HCM patient	22.2%	N/A	N/A	ZJ (E1b)	N/A
MT-TH	m.12192G>A	L3e1b2, G2a5, H45a, B5b1a2	0.24	nt.59 (T-loop)	R/MIMC	7.5%	N/A	Not Conserved	none	N/A
MT-ND5	m.12477T>C	7 haplogroups	0.61	p.S47 =	R/pos. HCM susceptibility	97.5%	N/A	N/A	XS(H)	N/A
MT-ND5	m.13135G>A	K2b1a + 12 other haplogroups	0.97	p.A267T	R/pos. HCM susceptibility	10.0%	Not Conserved (4)	N/A	ZX (K2b1), XQ (H)	0.13

Three variants, identified in this cohort, have previously been associated with HCM susceptibility: *MT-RNR1*: m.869C>T, *MT-ND5*: m.12477T>C, p.47S = and *MT-ND5*: m. G13135A, p.A267T [54]. Here they were sorted out as they did not fulfil the positive criteria due to their high prevalence (Table 4). However, none of these variants have been given the status “Confirmed” in MitoMap database and our results support that they are not pathogenic.

Dimaruro and Schon have presented four canonical characteristics that support pathogenicity of a mtDNA variant. The variant must affect an evolutionary conserved and functionally important site, pathogenic mutations are often heteroplasmic, the level of heteroplasmy should segregate with the degree of the severity of the disease in maternally related family members and, finally, the variant should not be present in an ethnically matched control population [41].

That the mutation should be absent in ethnically matched controls is evident. We did, however, use the 24187 whole mtDNA sequences available at GenBank, at time of search as control population. These are not ethnically matched and often there is no demographic or clinical information available. Thus, we cannot know whether the matching sequence belongs to very young persons or undiagnosed HCM patients. This can be a source of error when studying HCM, where the disease development is age dependent with variable phenotypic presentation and penetrance.

Homoplasmy of a variant suggests that it is not pathogenic. However, mtDNA mutations causing HCM in adults with no multisystemic involvement are not expected to be severe and can therefore be homoplasmic, as homoplasmic mutations are not expected to cause clinical symptoms until after reproductive age [56]. In addition, homoplasmic mt-tRNA mutations tend to exhibit tissue-specificity [57]. Furthermore, a homoplasmic mutation can only produce a pathological phenotype in combination with environmental or genetics factors [58,59]. In previously reported cases of HCM caused by heteroplasmic mtDNA mutations, the individuals were <11 years of age with multisystem involvement [60–63], while cases of isolated HCM caused by a homoplasmic mutation have been found in affected and unaffected family members ranging from 5–90 years of age [64–66]. The significance of incomplete penetrance of homoplasmic mutations is illustrated by cases where severe homoplasmic mtDNA mutations have been compensated for by genetic and epigenetic factors [67,68].

*In silico* tools for predictions of the functional impact of amino acid changes use predictive algorithms utilizing evolutionary information, physio-chemical characteristics and structural information to calculate the probability of the effect of non-synonymous variants. The prediction success have been found to be gene dependent and the accuracy of the predictions are improved when consensus are obtained from multiple algorithms [69,70]. The resulting Index6 has a sensitivity of 80%, and a specificity of 98% with a cut-off at 0.75. This represents an outperformance of all the individual prediction servers. However, as the prediction scores were obtained using a small training set of well-established mtDNA variants and the algorithms are not optimized specifically to mitochondrial proteins, the algorithm was supplemented with a criterion stating that the conservation score should be ≥8.

Only one variant (*MT-CYB*: p.C93Y; m.15024G>A) identified in this cohort fulfilled all the criteria to be considered potentially pathogenic. The variant has previously been described as a potentially modifying mutation based on molecular modelling, by our group, and as a potential modifier role in deafness expression in combination with other mutations [36,52]. The p.C93Y amino acid substitution is expected to partially disrupt protein function by altering the local protein conformation [36]. In addition, a putative disease-causing sarcomeric mutation has been identified in the proband, thus even through it fulfilled all the criteria for being considered potentially pathogenic it can at most be considered as a potentially modifying variant.

Pathogenic point mutations in the mt-tRNA genes are responsible for the majority of the mitochondrial diseases even though these genes only constitute 9% of the mtDNA [57].

The molecular mechanism of pathogenicity of mt-tRNA mutations includes prevention of amino acetylation, disruption of transcription factor binding, failed codon recognition and interference of post-transcriptional modifications and maturation [57]. A general characteristic feature of mt-tRNA mutations is a negative effect on mt-tRNA stability [71]. Differences in base-pairing, i.e. whether a wobble, Watson-Crick or mismatch may influence structural integrity of tRNAs [72].

The two tRNA variants remaining after filtering in this study, are located in MT-TF and MT-TC. The variant, mt-tRNA^Phe^: m.644A>G, is positioned in the acceptor stem changing 70A:3T Watson-Crick to a G:T wobble base pair. Two pathogenic homoplasmic mutations, m.3303C>T and m.3302A>G, located in the acceptor stem of mt-tRNA^Leu(UUR)^ both changes a Watson-Crick to G:T wobble. Both have the status of confirmed pathogenic in the MitoMap database (Table 5) [6].

**Table 5:** The previously “disease-associated” mt-tRNA variants.

Locus	rCRS position	Haplogroup	Freq GB (%)	Consequence	Heteroplasmy	Homoplasmy	Mitomap status	Conservation (MitoMaster)	Marmit-trna (Euarchontoglires)
MT-TL1	m. 3303C>T	None	0%	72C:1G > T:G wobble	+	+	Cfrm/MMC	82.5%	Not conserved
MT-TL1	m.3302A>G	None	0%	71A:2T > G:T wobble	+	-	Cfrm/MM	100.0%	Highly conserved
MT-TK	m.G8342A	None	0%	53G:61C > A:C mismatch	+	-	Reported/PEO and Myoclonus	62.2%	Not Conserved

The m.3303C>T mutation has a diverse clinical phenotype and symptoms ranging from sudden infant death syndrome to cardiomyopathy and skeletal myopathy in a 67 year old individual harbouring the mutation at homoplasmic level. The m.3302A>G in heteroplasmic form has been associated with mitochondrial myopathy [73–75]. The mechanism of pathogenicity has been suggested to be deficiency in the mt-tRNA maturation steps caused by reduced CCA addition to the 3´end by tRNA-nucleotidyltransferase and reduced 3´-tRNase cleavage resulting in accumulation of processing intermediates consisting of *MT-RNR2*, *MT-TL1* and *MT-ND1* mRNA (RNA 19) and concomitant reduced levels of mt-tRNA^Leu(UUR)^ [48, 74,76].

The, *MT-TC*: m.5772G>A, also identified in this cohort, is positioned in the T-stem.

The only confirmed pathogenic mutation in the mitomap database, located in the T-stem, that has been characterized, is the m.12315G>A (52G:62C WC > A:C mismatch, mt-tRNA^Leu(CUN)^) associated with the diseases Chronic Progressive External Ophthalmoplegia and Kearns-Sayre syndrome [6]. Functional characterization of m.12315G>A showed a decrease in aminoacylation, altered CCA addition to the 3´end by tRNA-nucleotidyltransferase, inhibiting post-modification and interaction with human mitochondrial elongation factor Tu [77]. The induced multiple effects on tRNA metabolism by this T-stem mutation is likely specific for tRNA^Leu(CUN)^ as mismatch mutations at similar positions are accepted in other mt-tRNAs, this supports that polymorphic and pathogenic mt-tRNA variants are randomly distributed with no basic unifying features supporting, with the exception that most pathogenic mutations, affect highly conserved nucleotides [71]. In the case of the two mt-tRNA mutations identified in this cohort, it is difficult to predict if these have any effect on the tRNA metabolism and as putative disease causing sarcomeric mutations have been identified in the probands, these variants are classified as variants of unknown significance.

In conclusion, despite the considerable number of variants identified in this study we find that private mtDNA mutations are a very rare—if at all—cause of isolated HCM in adult individuals with no systemic involvement.

## Limitations

The study is limited by having been performed in a single population and by the small size, n = 87, of the HCM cohort. The use of the GenBank database as control population is also a limitation as the sequences are not ethnically matched and frequently neither demographic nor clinical information is available. Furthermore, the sequencing of mtDNA using DNA isolated from blood, may not represent the sequence of mtDNA in cardiac tissue.
